# Detection of Multiple Stationary Humans Using UWB MIMO Radar

**DOI:** 10.3390/s16111922

**Published:** 2016-11-16

**Authors:** Fulai Liang, Fugui Qi, Qiang An, Hao Lv, Fuming Chen, Zhao Li, Jianqi Wang

**Affiliations:** School of Biomedical Engineering, Fourth Military Medical University, Xi’an 710032, China; liangfulai@fmmu.edu.cn (F.L.); qifgbme@outlook.com (F.Q.); anqiang900903@163.com (Q.A.); fmmulvhao@fmmu.edu.cn (H.L.); fumingfmmu@outlook.com (F.C.); lizhaofmmu@fmmu.edu.cn (Z.L.)

**Keywords:** vital sign, detection, ultra-wideband (UWB), multiple-input and multiple-output (MIMO), radar

## Abstract

Remarkable progress has been achieved in the detection of single stationary human. However, restricted by the mutual interference of multiple humans (e.g., strong sidelobes of the torsos and the shadow effect), detection and localization of the multiple stationary humans remains a huge challenge. In this paper, ultra-wideband (UWB) multiple-input and multiple-output (MIMO) radar is exploited to improve the detection performance of multiple stationary humans for its multiple sight angles and high-resolution two-dimensional imaging capacity. A signal model of the vital sign considering both bi-static angles and attitude angle of the human body is firstly developed, and then a novel detection method is proposed to detect and localize multiple stationary humans. In this method, preprocessing is firstly implemented to improve the signal-to-noise ratio (SNR) of the vital signs, and then a vital-sign-enhanced imaging algorithm is presented to suppress the environmental clutters and mutual affection of multiple humans. Finally, an automatic detection algorithm including constant false alarm rate (CFAR), morphological filtering and clustering is implemented to improve the detection performance of weak human targets affected by heavy clutters and shadow effect. The simulation and experimental results show that the proposed method can get a high-quality image of multiple humans and we can use it to discriminate and localize multiple adjacent human targets behind brick walls.

## 1. Introduction

The radar technology is widely used in noncontact medical measurement, through-wall surveillance and post-disaster rescue operation [1,2,3,4]. According to [5], the IEEE maximum permissible exposures are 2 W/m^2^ for frequencies between 30 and 400 MHz. It ramps up from 2 to 10 W/m^2^ between 400 and 2000 MHz. For frequencies greater than 2000 MHz, the maximum permissible exposure is 10 W/m^2^. The electromagnetic radiation from the ordinary microwave radar sensors poses no safety threat [6]. The electromagnetic (EM) waves transmitted by radar can penetrate non-metallic obstacles (such as the walls and the ruins) and detect the vital signs in a standoff distance [7]. Therefore, life detection based on radar has become a hot research topic in recent years.

Nowadays, most applied radar techniques for human detection are primarily aimed at single human target detection and have made remarkable progress. However, in a real ruin environment, there always exist multiple buried survivors. The detection and localization of multiple human targets is urgently required since it can remarkably improve the efficiency of the post-disaster rescue. However, automatic detection of multiple stationary humans is a tougher problem due to the mutual interference of multiple human bodies. The mutual interference mainly includes the strong sidelobes of multiple humans and the shadow effect. Due to individual difference, reflection intensity of different humans has significant difference. Sidelobes of human target with strong reflection will submerge the signal of the adjacent human targets with weak reflection. 

Humans are no longer point targets in high-resolution imaging radar applications. For example, the frequency range of a common high resolution radar system is usually 0.5–3 GHz or even larger. If the frequency band is 2 GHz, the range resolution is 7.5 cm. However, the size of a common human is 1.8 m × 0.5 m × 0.35 m [8]. In this case, the human will occupy several resolution units. Considering the relationship between human size and resolution, human must be considered as an extended target, which will obscure the EM propagation. Thus, the EM waves, which should irradiate a certain human and reflected back to receiving element, will be partly blocked by the nearby humans. Therefore, the signal reflected from the distant humans will be weakened. We call the above phenomenon the shadow effect [9]. If the above interactions are misconducted, the leakage alarm rate will increase when there exist multiple humans in the detection region. The secondary damage may occur in the actual post-disaster rescue scenes. Much research effort has been made to solve the multiple stationary humans detection problem. 

Continuous wave (CW) radars are by far the most popular platform of stationary human detection, as they require relatively small hardware expense. Zhou et al. [10] developed a generalized likelihood ratio test (GLRT) for CW radar to distinguish between the presences of 2, 1 or no subjects. However, the drawback of CW radar is that they do not allow the localization of the humans and will lead to increased difficulty in interference suppression and multiple stationary humans discrimination.

Further performance improvements can be achieved by the ultra-wideband (UWB) radar. UWB waveform provides high range resolution ability, and thus has the potential to determine the distance of humans with high accuracy [11,12]. This allows accurate localization of the breathing subject and tracking of the small movements of the diaphragm during breathing [13]. In [14], UWB impulse radar was used to monitor the breathing rates of two subjects through a cement wall. Wang et al. [15] proposed a logarithmic method (LM) utilizing the phase variation of the reflected pulses caused by the periodic thorax displacements to monitor multiple subjects at low power consumption. The stepped frequency continuous wave (SFCW) is a new form of UWB radar waveform, which transmits a series of discrete frequencies in a stepwise manner, covering the radar bandwidth in the time domain to realize the UWB. The SFCW radar technology is superior to the time domain impulse radar for its high reliability and relative easy implementation. Liu et al. [16] used SFCW radar to detect vital signs from a human subject under laboratory conditions. Cardiologic signals can be achieved when the human subject was in the line of sight. However, these UWB systems use a single input and single output (SISO) channel, in which only range profile image of humans from single sight angle can be attained. For the applications of non-line-of-sight (NLOS), the detection performance of SISO degrades [16]. Meanwhile, three-dimensional space information of vital signs is projected onto the range dimension, which made it difficult to mitigate the mutual interference between multiple human bodies due to the aliasing problem. These challenges constrain the usage of SISO UWB radar in multiple stationary humans detection.

The radar system with multiple receiving channels, which can be termed as single-input multiple-output (SIMO), is used to achieve further improvement [17,18]. SIMO radar systems fuse the information from multiple channels to improve the detection performance. Akiyama et al. [19] used a system with one transmitting antenna and four receiving antennas to improve the signal-to-noise ratio (SNR) with correlation processing. Liu et al. [18] demonstrated that the SIMO radar systems have the ability to resolve multiple sources and obtain the angle-of-arrival (AOA) of multiple human targets.

Multiple-input and multiple-output (MIMO) radar is a special type of multiple channels radar which emerged in recent years. The MIMO array with M transmitting elements and N receiving elements can obtain a virtual aperture with M × N virtual transceivers, which greatly reduces the weight and cost of the radar system. MIMO radar echo data can be decomposed as the data from multiple SIMO radar system [20], since the MIMO radar system can attain and use information from more sight angles. UWB MIMO radar combines the high range resolution property of the UWB signaling with the directional resolution property of the multiple antenna elements, so it has the ability of two-dimensional high-resolution imaging [21]. Compared with the synthetic aperture radar (SAR), the UWB MIMO systems can get the multiple sight angles of target simultaneously, and the high resolution image sequence can be attained to describe the variation of the scenario. These advantages of the UWB MIMO radar have already attracted interests of researchers. It has been used for through-the-wall imaging of building structure surrounding the humans [22] and indication of moving human targets [9]. Salmi et al. [23] validated the performance of localizing a test subject and tracking his breathing under ideal conditions. Takeuchi et al. [24] localized survivors using ground-penetrating radar (GPR) with two-dimensional array antenna. However, multiple stationary humans detection is not discussed in these literatures.

In this paper, a UWB MIMO radar system is implemented and a novel signal processing method is proposed to improve the detection performance of multiple stationary humans. The stepped frequency continuous wave (SFCW) waveform is adopted to form ultrawide band and high range resolution capacity. As to the problem of the mutual interference among multiple humans, we propose a vital-sign-enhanced imaging algorithm. On one hand, this algorithm fully utilizes two-dimensional high-resolution imaging of UWB MIMO radar to isolate the human bodies and clutters in space, and thus the mutual interference will be mitigated. On the other hand, the mutual interference can be further suppressed by enhanced imaging. Then, a high resolution vital-sign-enhanced image sequence is formed. Aiming at the detection problem in low signal-to-clutter ratio (SCR) and shadow effect of multiple humans, preprocessing is firstly adopted to improve SCR of vital signs, and further an automatic detection algorithm is realized by using constant false alarm rate (CFAR), morphological filtering and clustering. Via testing the local contrast of the image, weak human targets influenced by clutters and shadow effect can be detected by CFAR. The shape and size features of the human target are utilized by morphological filtering and clustering to reduce the false alarms. The simulation and experimental results show that the proposed method can get high resolution images of multiple humans and accurately detect multiple humans even the targets are adjacent to each other. Two-dimensional localization of the subjects can also be precisely estimated by the proposed method.

The paper is structured as follows. Section 2 builds the vital signs model of UWB MIMO radar. Section 3 describes the proposed detection method. Section 4 gives the simulation results. Section 5 gives a brief description of the UWB MIMO radar system and illustrates the measurement results. Concluding remarks are given in Section 6.

## 2. Vital Signs Model of UWB MIMO Radar

When EM waves emitted from transmitting channel illuminates the human body, part of them will be reflected and received by the receiving channels. Due to respiration, the chest cavity expands and contracts periodically, so the round-trip distance d(τ) varies periodically around the nominal distance *d*_0_ accordingly. Considering the monostatic scattering and the line-of-sight situation, the human chest wall movement caused by respiration is

(1) $$d(\tau )=d_{0}+d_{b}\sin(2\pi f_{b}\tau )$$

where τ represents the slow time which corresponds to the acquisition time of each range profile. The range profile represents the projection of the human target scattering centers on the radar line of sight. *d_b_* is the amplitude of the chest wall displacement caused by respiration and $f_{b}$ is the respiration frequency. 

**Figure 1:**
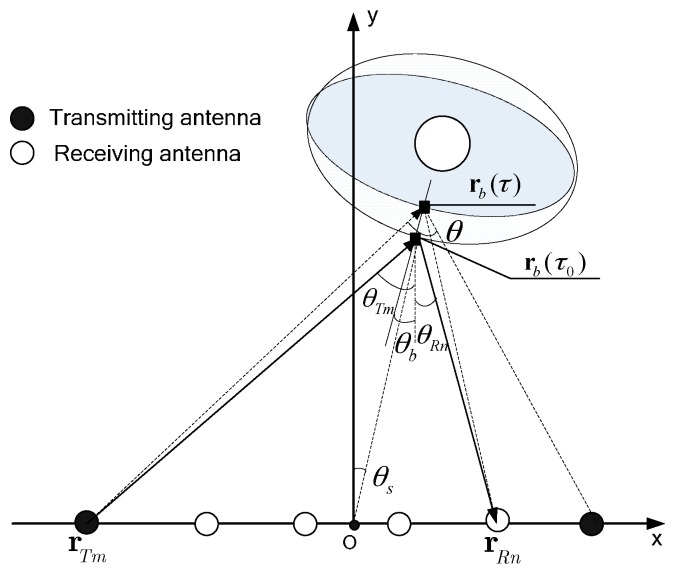
Model of vital signs in UWB MIMO radar.

For UWB MIMO radar, the transmitting antennas and receiving antennas are separately placed, so the bistatic model of vital signs should be considered. Figure 1 shows the propagation procedure of the incident EM waves transmitted by the *m*-th transmitting antenna to the target and the scattered EM waves of human body received by the *n*-th receiving antenna. The rectangular coordinates are built as Figure 1, where *x* and *y* denote the cross range and range direction, respectively. The origin is set to be the center of linear antenna array for convenience. For simplicity r represents the position vector of the subject with the form of $r^{T}=[x,y,z]$, where *z* is the height coordinate. Assuming that the *m-*th transmitting antenna locates at $r_{Tm}$, the *n-*th receiving antenna locates at $r_{Rn}$, the chest locates in $r_{b}(\tau )$ and the human body is an ideal ellipsoid, the round-trip range of the vital sign is

(2) $$\begin{array}{l} D_{m,n}(\tau )=\left|r_{Tm}-r_{b}(\tau )\right|+\left|r_{Rn}-r_{b}(\tau )\right|\\ \;=d_{0}+\Delta d(\tau )\\ \;\approx d_{0}+d_{b}[\cos(\theta_{Tm}-\theta_{b})+\cos(\theta_{Rn}+\theta_{b})]\sin(2\pi f_{b}\tau ) \end{array}$$

where

(3) $$d_{0}=\left|r_{Tm}-r_{b}(\tau_{0})\;\right|+\left|r_{Rn}-r_{b}(\tau_{0})\;\right|$$

$\theta_{Tm}$, $\theta_{Rn}$ and $\theta_{b}$ denote the aspect-angles of the transmitting antenna, the receiving antenna and the normal direction of chest, respectively. |·| denotes the vector length.

Equation (2) shows that the displacement induced by the respiration movement is a function of the bi-static angles and the attitude angle of the human body. Affected by $\theta_{b}$ it is possible to be non-line-of-sight case for some channels. While benefiting from multiple sight angles of UWB MIMO radar, the received echo may be approximately line-of-sight in some channels. Therefore, compared with SISO radar, UWB MIMO radar is not so sensitive in the detection of non-line-of-sight human target.

t is the fast time which represents the time axis associated with range along each range profile. It can be thought orthogonal to the slow time dimension. Let $s_{T}(t)$ be the transmitted signal. The received signal from the *m*-th transmitting antenna and the *n*-th receiving antenna can be expressed as

(4) $$s_{m,n}(t,\tau )=\sum_{p=1}^{P}s_{T}(t)⊗h_{p}(t)⊗\delta (t-\frac{D_{m,n,p}(\tau )}{c})\;+\sum_{q=1}^{Q}s_{T}(t)⊗h_{q}(t)⊗\delta (t-\frac{d_{m,n,q}(\tau )}{c})$$

where *c* is the speed of flight, δ(⋅) is the Dirac function, ⊗ denotes the convolution operation, $h_{p}(t)$ is the impulse response of the *p-*th vital sign, $h_{q}(t)$ is the impulse response of the *q-*th clutter including directive wave, coupling clutter and stationary or non-stationary clutter, $D_{m,n,p}(\tau )$ is the round-trip distance between the *p-*th human and the *m-*th transmitting antenna and the *n-*th receiving antenna, $d_{m,n,q}(\tau )$ is the round-trip distance between the *q-*th clutter and the *m*-th transmitting and the *n*-th receiving antenna.

If the fast time is sampled with the sampling interval *δ_T_* and each range profile contains *K* samples, the sampling interval of slow time is equal to *δ_T_* × *K* which is the processing time of one range profile. For the UWB MIMO radar, if *M* transmitting elements sequentially emit SFCW signals, the sampling interval of slow time will be *δ_T_* × *K* × *M.* Thus, the MIMO radar which sequentially emits signals sacrifices the sampling frequency along slow time domain to get low-complexity radar system design. The discrete signal of each channel can be expressed as two-dimensional matrix

(5) $$S_{m,n}[k,l]=h_{m,n}[k,l]+c_{m,n}[k,l]\;+w_{m,n}[k,l]$$

where k=0,1, . . . , K−1 is fast time index and l=0,1, . . . , L−1 represents the slow time index. $h_{m,n}[k,l]$ is the response of vital signs, $c_{m,n}[k,l]$ is the response of clutters and $w_{m,n}[k,l]$ is the additive noise. The received data set S will be a three-dimensional matrix, which contains fast time, slow time and equivalent channel data, respectively. The information of respiration movement is contained in the received data S and can be utilized in multiple humans detection.

## 3. Automatic Detection Method

**Figure 2:**
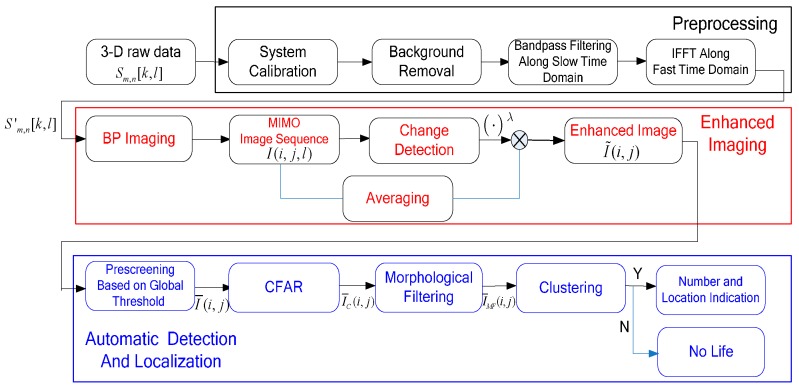
Flowchart of automatic detection method.

In this section, a novel detection method based on UWB MIMO radar is proposed to detect vital signs of multiple stationary humans automatically. As illustrated in the flowchart shown in Figure 2, the detection method consists of three main procedures including preprocessing, enhanced imaging and automatic detection and localization.

### 3.1. Preprocessing

The preprocessing procedure is first applied to the raw data obtained in each channel before enhanced imaging. System calibration, background removal, bandpass filtering along slow-time domain for SNR improvement and inverse fast Fourier transform (IFFT) along fast time domain are included in the preprocessing step.

The aim of system calibration is to guarantee the coherence between multiple channels. The calibration data are collected by the following two manners. One manner is to set up a reference channel. The other manner is to use a point-like object, such as the trihedral, as the reference target, and collect the data of each channel in empty background as the calibration data. In this paper, the phase of calibration data collected by the above two manners is used to calibrate the incoherence.

The background components can be seen as strong and static clutters. Therefore, the vital signs can be enhanced by change detection (CD) [25]. Background removal is one simple method of CD. A simple way to remove background is subtracting the mean value over the slow-time window as

(6) $${S^{\prime }}_{m,n}[k,l]=S_{m,n}[k,l]-\frac{1}{L}\sum_{l=0}^{L-1}S_{m,n}[k,l]$$

The respiration frequency is about 0.2–0.3 Hz, which is much lower than the slow time pulse repetition frequencies (PRF) of the UWB MIMO echo. As a result, the raw data is severely oversampled and lots of clutters are induced into the echo. According to the prior knowledge of respiration frequency, bandpass filtering along slow time is used to eliminate the clutters and harmonic components with high frequency and ultra-low frequency components. 

For SFCW radar, the echo can be viewed as the frequency response of the target. Thus, the IFFT is performed to get high resolution range profile (HRRP). A frequency window, such as hanning window and hamming window, is added to suppress the range sidelobes.

### 3.2. Enhanced Imaging of Multiple Vital Signs

#### 3.2.1. BP Imaging

The UWB MIMO radar is usually performed in near-field and bistatic mode. Among the image formation methods, the back-projection (BP) imaging algorithm, which is well-known for its high precision and simplicity and well adaptation to near-field imaging, is employed in this paper as the basic imaging method.

For certain slow-time sample $l_{0}$, the BP image of the MIMO array with *M* transmitting channels and *N* receiving channels can be obtained by the coherent sum of the images of *M* SIMO arrays as

(7) $$I(x,y,l_{0})=\sum_{m=1}^{M}I_{m}(x,y,l_{0})$$

where x and y are the range coordinate and cross-range coordinate of the image grid, $I_{m}(x,y,l_{0})$ is the image formed via the *m*-th SIMO array and can be represented as

(8) $$I_{m}(x,y,l_{0})=\sum_{n=1}^{N}h_{Tm}(x-x_{Tm},y)h_{Rn}(x-x_{Rn},y){S^{\prime }}_{m,n}(t,l_{0})\delta (t-\frac{\sqrt{y^{2}+{\left(x-x_{Tm}\right)}^{2}}+\sqrt{y^{2}+{\left(x-x_{Rn}\right)}^{2}}}{c})$$

where $h_{Tm}(x-x_{Tm},y)$ and $h_{Rn}(x-x_{Rn},y)$ are the window functions of transmitting element and receiving element to calibrate the antenna directional pattern and control the aperture shape.

The range resolution of BP image is determined by

(9) $$\rho_{R}=\frac{c}{2B}$$

where B is bandwidth of UWB MIMO radar system. The cross-range resolution is determined by [26]:

(10) $$\rho_{C}=\frac{0.886\lambda_{c}}{4\sin(\theta /2)cos(\theta_{s})}$$

where $\lambda_{c}$ is the wavelength of the center frequency, and θ and $\theta_{s}$ are the accumulated and squint angles between the array and the target, respectively, as depicted in Figure 1. Wider bandwidth, higher center frequency, shorter range, and longer array result in finer $\rho_{R}$ and $\rho_{C}$. However, considering the portability and penetrability requirement of the radar, the array length and the center frequency of the UWB MIMO radar system are strictly restricted, accompanied with the limited range and cross-range resolution.

#### 3.2.2. Vital Signs Enhancement Based on CD

The human body can be seen as a complex extended target with certain size and shape [11]. The reflection of the torso is the strongest, and it has large area and strong sidelobes in BP image. On one hand, the torso will interfere with the arms and legs in the image formation, which makes it difficult to get a silhouette image by the conventional imaging algorithms. On the other hand, strong sidelobes of torsos interfere with each other, which lead to the deteriorated imaging quality of multiple humans. In addition, strong environmental clutters make the detection of multiple stationary humans more difficult. Although nominal resolution of UWB MIMO image is high, the conventional BP image cannot satisfy the requirement of detection and localization of multiple humans.

In through-barrier applications, heavy stationary clutters are usually removed by coherent or noncoherent CD, based on the fact that there always exist features of humans characterized by respiration, movements of limbs, etc. [27,28]. The motions of limbs and respiratory movement are prominently distinguishable features between humans and environmental clutters, which can facilitate the human detection. For a stationary human, except for chest fluctuation caused by respiration motion and micro movement of limbs, the other parts of body can be seen relatively stationary. After CD processing, the vital signs are enhanced and stationary body parts are suppressed. The strong sidelobes of human torso are suppressed effectively. Thus, the equivalent distances between multiple humans and environmental clutters become larger in image after CD. 

For a trapped human, the limbs are relatively stationary, so the vital signs can be utilized are only respiration signal. The vital signs can be approximated as sinusoidal signal with small amplitudes. For a standing human, the micro motions of the limbs are inevitable even we try to keep stationary. These motions will form signal component with high reflectivity in received echo, but they varies randomly along the slow time. Delay line canceller based on two frames or several frames is unsuitable to extract the random signal [25].

The CD algorithm should consider the above two conditions. In this paper, we adopt CD with the form of variation for its satisfaction with the above requirement and easy implementation to integrate motion information during long time

(11) $$I_{1}(i,j)={\sum_{l=0}^{L-1}\left|I(i,j,l)-\sum_{l=0}^{L-1}\left|I(i,j,l)\right|/L\right|}^{2}$$

where L is the total number of the slow time samples in the attained UWB MIMO image sequence. In Equation (11), UWB MIMO image sequence is projected onto range-cross-range plane by variation along slow time. For each pixel sequence, the change part will be reserved and accumulated. The stationary part will be seen as mean value and removed by variation operation. Therefore, the vital signs with micro-motion from multiple humans are enhanced. However, some weak non-stationary interference in the environment will also be enhanced. This may cause false alarm in the detection procedure. 

Weak interference with micro motions can be suppressed by average of BP image sequence along the slow time domain as follows

(12) $$I_{2}(i,j)=\sum_{l=0}^{L-1}\left|I(i,j,l)\right|/L$$

The operation of Equation (12) is actually a simple low pass filter, which can eliminate the high frequency micro displacement, and thus achieve the purpose of suppressing the micro motion component. The enhanced image of the multiple humans are given as [29]

(13) $$\tilde{I}(i,j)=I_{1}{\left(i,j\right)}^{\lambda }\cdot I_{2}(i,j)$$

where λ is the relaxation factor which controls the enhancement of vital signs. In this paper, λ is selected to be 1 according to the practical experience.

### 3.3. Automatic Detection and Localization of Multiple Stationary Humans

#### 3.3.1. Prescreening Based on Global Threshold

In order to avoid false alarms due to weak noise generated in the process of calculating the local contrast of the image in CFAR, prescreening based on global threshold should be performed first to process the near-zero value pixels. This process is represented as Equation (14): the pixel values smaller than $T_{g}$ will be replaced by zero; otherwise, the pixels keep the original values.

(14) $$\bar{I}(i,j)=\{\begin{array}{c} \tilde{I}(i,j),\tilde{I}(i,j)\geq T_{g}\\ \;0,\;\tilde{I}(i,j)<T_{g} \end{array}$$

where $T_{g}$ is the global threshold determined by all the pixel values of the enhanced image as

(15) $$\frac{Number\left[\tilde{I}(i,j)<T_{g}\right]}{Number\left[\tilde{I}(i,j)\right]}=\gamma$$

where Number[·] is the operation of computing the elements number which meets the given condition in braces. γ is determined according to the experimental results and the value is selected to be 0.1 in our experiment. Thus, the pixels whose values are the smallest 10% are set to be zero.

#### 3.3.2. CFAR Detection

Although the vital signs are notably enhanced by the above processing, there still exist heavy clutters in complex scenarios, e.g., detection of trapped survivors in the ruins. Influenced by the shadow effect, reflection of some humans may be much weaker than the other humans. CFAR is adopted to automatically detect multiple vital signs with large magnitude difference in low SCR scenarios. 

**Figure 3:**
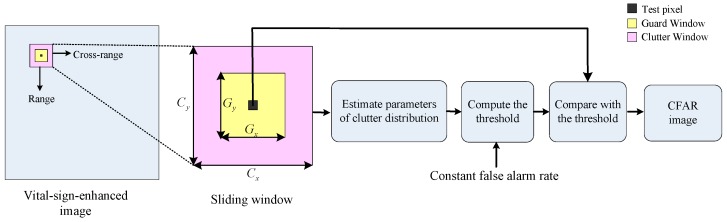
Architecture of CFAR based on vital-sign-enhanced image.

As is shown in Figure 3, CFAR uses a 2-D sliding window to scan all pixels in the vital-sign-enhanced image to search suspected vital signs. The pixel to be detected locates at the center of the sliding window. The sliding window includes the guard window and the clutter window. As Figure 3 shows, $G_{x}$ and $G_{y}$ are the cross-range and range dimensions of guard window, respectively. $C_{x}$ and $C_{y}$ are cross-range and range dimensions of the clutter window. The guard window is designed to be overlaid on the vital sign. Clutter window is designed to be superimposed on local background. Generally, vital sign spreads in range and cross-range dimensions because of the multipath reflections of each body part as well as the propagation, attenuation, and reflection of the EM waves inside the body. Hence, guard window is a buffer between the tested pixel and clutter window to ensure the vital sign is not captured by the clutter window as the clutter background.

According to the size of human chest, $G_{x}$ and $G_{y}$ are set to be:

(16) $$\begin{array}{l} G_{x}\approx odd\left\lceil \frac{\max(K_{chest},d_{chest})+L_{arm}/2}{x_{res}}\right\rceil \\ G_{y}\approx odd\left\lceil \frac{\max(K_{chest},d_{chest})+L_{arm}/2}{y_{res}}\right\rceil \end{array}$$

where odd⌈x⌉ denotes the smallest odd integer larger than *x*, $x_{res}$ and $y_{res}$ denote the grid width in cross-range and range dimension, $d_{chest}$ and $K_{chest}$ represent the prior knowledge of the thickness and width of human chest which denote the influence induced by penetration and multiple reflections within the human body, $L_{arm}$ is the length of the arm. Considering that the stationary human body has micro body movement, the reflection of arms and legs maybe the strongest in this situation. Therefore, the size of guard window is expended by half length of the arm.

Clutter window is defined by $C_{x}\approx odd\left\lceil G_{x}+\frac{D_{H}}{x_{res}}\right\rceil ,C_{y}\approx odd\left\lceil G_{y}+\frac{D_{H}}{y_{res}}\right\rceil$, where $D_{H}$ is the extended distance considering the interference between two human bodies. Usually, when the value of $D_{H}$ is selected to be large, the pixel number used for distribution parameter estimation of the clutter is large. Thus, the estimation of distribution parameter is relatively accurate. However, in order to eliminate the influence of nearby human body, $D_{H}$ should be smaller than the distance between two human targets. In most real applications, even the adjacent humans are also separated with an interval of larger than 0.1 m. Thus, in our processing procedure, $D_{H}$ is set to be 0.2 m.

The statistical distribution model of the surrounding clutter in the clutter window is then estimated. For the real data, the probability density function (PDF) of clutter is unknown. The best probability density function for the corresponding clutter was determined by non-parameter histogram method [30]. The histograms of background image are firstly constructed. Then, classic PDF models (Gaussian distribution, gamma distribution, Weibull distribution, lognormal distribution, etc.) are compared with the obtained histogram. The model with the minimized mean squared error is chosen as the PDF of clutter. It was found that background clutters are best approximated by the lognormal distribution, which exhibits non-Gaussian characteristic.

The threshold is calculated with a given false alarm rate (FAR) based on the estimated clutter model. The detected pixel is determined to be part of a human target when the amplitude exceeds the threshold, which can be defined as [31]:

(17) $$\{\begin{array}{c} \bar{I}(i,j)\geq T_{CFAR}\;(i,j)\;\mathrm{belongs}\;\mathrm{to}\;\mathrm{vital}\;\mathrm{sign}\\ \bar{I}(i,j)<T_{CFAR}\;(i,j)\;\mathrm{does}\;\mathrm{not}\;\mathrm{belong}\;\mathrm{to}\;\mathrm{vital}\;\mathrm{sign} \end{array}$$

where $T_{CFAR}$ denotes the threshold of CFAR.

#### 3.3.3. Morphological Filtering

Mathematical morphology is a well-known nonlinear image processing methodology based on the application of lattice theory to spatial structures. Along with the development of morphological theory, morphological image processing method has gradually become a new trend in image processing field and a favorable tool in weak target detection [32]. The basic morphological operations include erosion, dilation, opening and closing. We can obtain some important compound operations with different characteristics by combining them. In this paper, morphological filtering is used to eliminate the irrelevant object which is a different size from vital signs. 

The opening Top-Hat used to subtract clutters with large size is defined as follows [33]:

(18) $${\bar{I}}_{OTH}=\bar{I}-\bar{I}\circ g$$

where “∘” denotes the morphological opening including erosion operations followed by dilation operations, and *g* is the morphological structuring element. The pixels with negative amplitude are set to be zero,

(19) $${\bar{I}}_{OTH}(i,j)=\{\begin{array}{c} {\bar{I}}_{OTH}(i,j)\;{\bar{I}}_{OTH}(i,j)\geq 0\\ \;0\;{\bar{I}}_{OTH}(i,j)<0 \end{array}$$

The opening operation is used to subtract clutters of small size:

(20) $${\bar{I}}_{MF}={\bar{I}}_{OTH}\circ s$$

where *s* is the morphological structuring element to subtract small clutters. 

In this paper, both *g* and *s* are flat, disk-shaped structuring elements but different in radius size. The sizes are determined by combination of experimental data analysis and the prior knowledge of human size.

#### 3.3.4. Clustering

In CFAR image, a small number of scatters typically dominate the target returns and these bright pixels cannot be connected into a region, so the close-by target pixels are clustered in target-size regions via a clustering algorithm, e.g., the K-means clustering method [34]. Then a chip around each cluster center is taken out and considered as a suspected target.

Assuming that the image ${\bar{I}}_{MF}\left(i,j\right)$ contains *P* vital signs with *P* centroids $\left\{\mu_{1},\mu_{2},\cdots ,\mu_{P}\right\}$, *V* non-zero points $\Phi =\left\{\varphi_{1},\varphi_{2},\cdots ,\varphi_{V}\right\}$ are surrounded around the *P* centroids. An iterative procedure is used to identify the centroids of suspected vital signs as follows:

**Step** **1:** The strongest non-zero pixel is chosen as the initial cluster center $u_{1}$. The range between the *i*-th pixel and the cluster center is computed as $D_{i1}={\left\|u_{1}-\varphi_{i}\right\|}_{2}$. The location of the centroid is then updated as $u_{1}^{\prime }=\frac{1}{m_{1}}\sum_{i=1}^{m_{1}}\varphi_{i}$ using the pixels of nearby non-zero pixel satisfying the condition of $D_{i1}<d_{c}$, where $m_{1}$ is the pixel number used in the updating and $d_{c}$ is the clustering radius. According to the prior information of human body size, $d_{c}$ is set to be 0.5 m in this paper. The pixels satisfying $D_{i1}<d_{c}$ are categorized into cluster $C_{1}$.
**Step** **2:** $\Phi_{1}$ is obtained by removing the pixel in $C_{1}$ from Φ. The strongest pixel in $\Phi_{1}$ is chosen as the initial cluster center $u_{2}$. The centroid is updated similar as Step 1, and cluster $C_{2}$ is obtained.
**Step** **3:** Repeat Step 1 and Step 2 until all pixels in Φ are categorized into the according clusters. $N_{c}$ clusters $\left\{C_{1},C_{2},\cdots ,C_{N_{c}}\right\}$ and $N_{c}$ corresponding cluster centroids $\left\{u_{1}^{\prime },u_{2}^{\prime },\cdots ,u_{N_{c}}^{\prime }\right\}$ are obtained.
**Step** **4:** Compute the number of pixels $N_{p}$ in each cluster. If $N_{p}<N_{T}$, the cluster is removed. $N_{T}$ is the smallest pixel number of the vital sign, and determined by real experiments. Thus, we get *P* clusters and *P* centroids $\left\{c_{1},c_{2},\cdots ,c_{P}\right\}$ in the final clustering results.

The number of life signs and the detailed vital information are automatically given by the results of the above clustering algorithm. If the number of clusters is zero, we decide that no life sign exists. If the number of clusters is not zero, the locations are given by the cluster centroid. The pixel sequences at the estimated locations are extracted and the respiration frequency is estimated by maximum magnitude of the spectrum.

## 4. Simulations and Results

The received echo reflected from the displacement of human chest for a UWB MIMO system was simulated with MATLAB 2013. The uniform linear MIMO array is composed of two transmitting elements and four receiving elements with interelement space of 0.5 m. The two transmitting antennas are settled on the leftmost side and rightmost side of the MIMO array. The transmitted signal was SFCW waveform with frequency range from 40 MHz to 4400 MHz. The stepped frequency interval is 5 MHz. The pulse repeated frequency (PRF) is about 110 Hz. The vital signs are simulated as ideal point targets with periodical sinusoidal displacement. In this simulation, the center of the MIMO array is set to be origin of the coordinate system. The range coordinates of three vital signs are 2 m, and the cross-range coordinates are −0.2 m, 0.2 m and 0.3 m, respectively. The vital signs are simulated in free space and their simulated respiration frequencies are 0.2 Hz, 0.3 Hz and 0.4 Hz, respectively.

**Figure 4:**
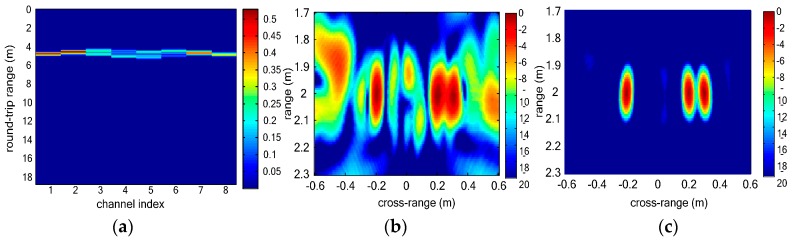
Simulated UWB MIMO image results of multiple vital signs: (**a**) range profile of each virtual channel; (**b**) BP image of three simulated vital signs; and (**c**) Vital-sign-enhanced image.

**Figure 5:**
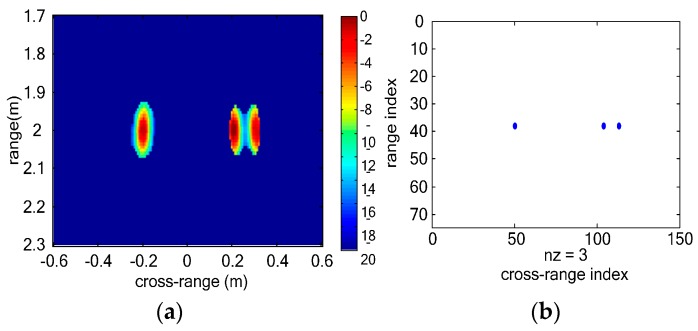
Simulation result of multiple vital signs: (**a**) CFAR result; and (**b**) clustering result.

As Figure 4a shows, not all the vital signs can be discriminated in range profile of each virtual channel because the range coordinates of three vital signs are similar. In Figure 4b, three vital signs can be discriminated. However, sidelobes of vital signs are strong and interference among multiple vital signs is serious. In vital-sign-enhanced image, the sidelobes are suppressed and thus the image quality of vital signs is much better. We can distinguish multiple vital signs even when the distance between the two vital signs is 0.1 m. Figure 5 shows the final detection results of simulated vital signs. In the clustering result, three vital signs are detected. The estimated range coordinates of all the three vital signs are 2.0040 m, and the estimated cross-range coordinates are −0.2000 m, 0.2240 m and 0.2960 m, respectively. The estimation error of the localizations is 0.004 m which is close to the size of the image grid.

## 5. Experiment and Results

### 5.1. Measurement Setup

**Figure 6:**
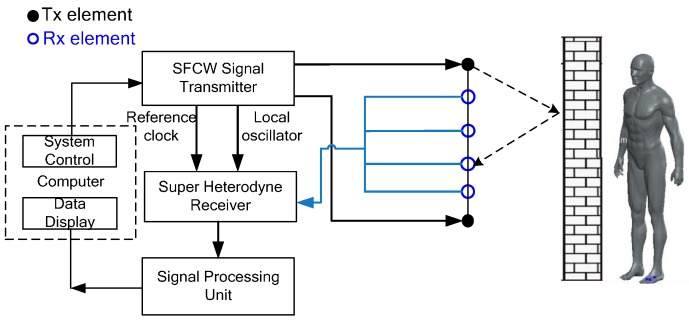
Configuration of UWB MIMO radar system.

The setup of UWB MIMO radar system is depicted in Figure 6. The linear UWB MIMO array consists of six uniformly distributed planar logarithmic spiral antenna with element space of 0.6 m. Two transmitting elements are located at the leftmost and rightmost end and the remaining elements are four receiving antennas. The transmitting and receiving antennas employ reversal polarization pattern. The two transmitting elements sequentially emit SFCW signals, sweeping from 40 MHz to 4.4 GHz at a frequency step of 5 MHz, while the receiving elements sample the scattered echoes simultaneously. Thus, each scan has eight T*/*R channels in total and the operation mode of each channel can be regarded as the same. The SFCW signal can be viewed as a complex signal representing the frequency response of the target echo which was measured sequentially at a set of discrete frequencies. Each scan takes 8.8 ms, at a frame rate of about 113 Hz.

In super-heterodyne receiver, the received radar echo is down-converted into intermediate-frequency (IF) signal, and then IF filtering and digital quadrature demodulation are performed to get the complex signals of vital signs. The parameters of the UWB MIMO radar are detailed in Table 1.

Both through-wall detection experiments of single and multiple stationary humans using UWB MIMO radar have been implemented in this paper. The subjects stand behind a brick wall (about 30 cm thick), and face the array, which is placed along the wall. The subjects stand still with normal breathing.

### 5.2. Experiment Results and Performance Analysis

(1) Through-wall detection of single stationary human

**Figure 7:**
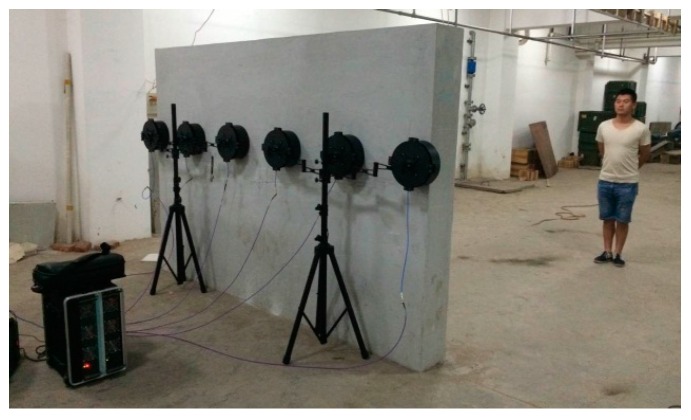
UWB MIMO radar system and the experimental scene.

**Figure 8:**
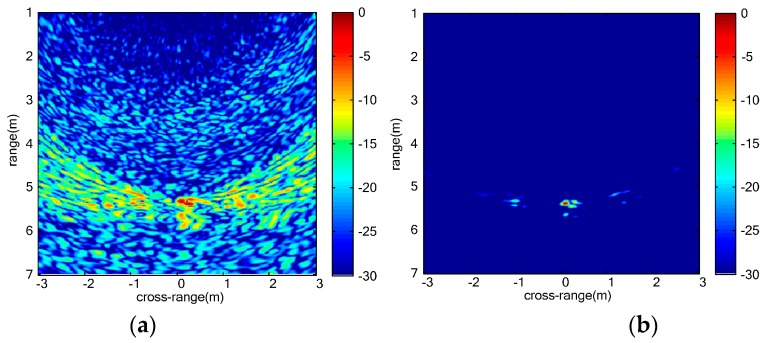
Images of single stationary human: (**a**) UWB MIMO image at certain slow time; and (**b**) vital sign-enhanced image.

**Figure 9:**
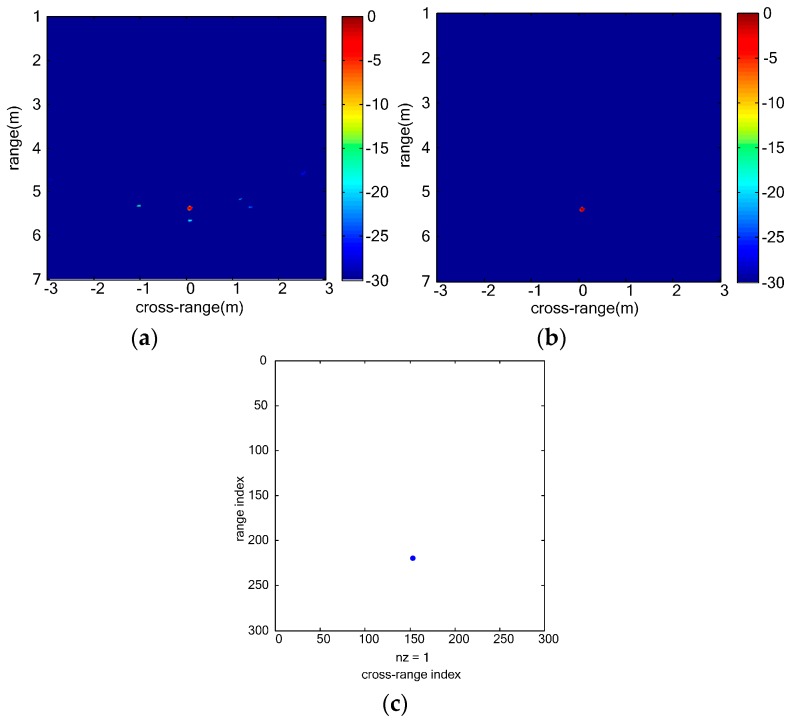
Detection results of single stationary human: (**a**) CFAR image; (**b**) morphological filtering result; and (**c**) clustering result of single stationary human.

The scenario of the single stationary human measurement is shown as Figure 7. The subject stands 3 m away from the center of the MIMO array. UWB MIMO image at a certain slow time frame is shown as Figure 8a. Strong sidelobes can be seen in Figure 8a, and thus false alarms will be easily produced. The sidelobes are effectively suppressed in vital-sign-enhanced image shown in Figure 8b, and the SCR of vital signs are effectively improved. The SCR is defined as:

(21) $$\mathrm{SCR}=10\mathrm{lg}\frac{P_{t}}{P_{c}}=10\mathrm{lg}\frac{\sum_{(i,j)\in \chi_{t}}{\left|I(i,j)\right|}^{2}/N_{t}}{\sum_{(i,j)\in \chi_{c}}{\left|I(i,j)\right|}^{2}/N_{c}}$$

where $P_{t}$ is the pixel power in the target region $\chi_{t}$, $P_{c}$ is the pixel power in the clutter region $\chi_{c}$, and $N_{t}$ and $N_{c}$ are the pixel number in $\chi_{t}$ and $\chi_{c}$, respectively. In this paper, $\chi_{t}$ and $\chi_{c}$ are determined by guard window and clutter window around the center of the vital signs described in Section 3.3. The SCR of one frame of MIMO image is 16.8882 dB and the SCR of the vital-sign-enhanced image attains 50.6309 dB. From this result, we can conclude that the clutters and mutual interference can be mitigated. The improvement of the SCR is beneficial to raise the detection performance. The detection results of single stationary person are given in Figure 9, where the false alarm rate is set to be 10^−6^. In the CFAR image shown in Figure 9a, the vital sign is a bright and circular spot. Some weak clutters with high local contrast are also detected as pixels of suspected targets. Because these clutters are different from the vital signs in their shapes and sizes, the morphological filtering can effectively subtract these false suspected targets as shown in Figure 9b. After these processing steps, we can easily discriminate one vital sign. The measured location of detected vital sign is about at 0.38 m and 5.06 m in range and cross-range axis which agrees with the measured values.

(2) Through-wall detection of multiple adjacent stationary humans

**Figure 10:**
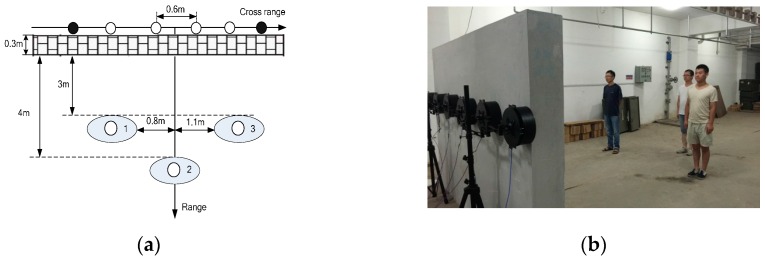
Experimental scene of multiple stationary humans detection: (**a**) schematic figure of the experimental scene; and (**b**) photograph of the experimental scene.

**Figure 11:**
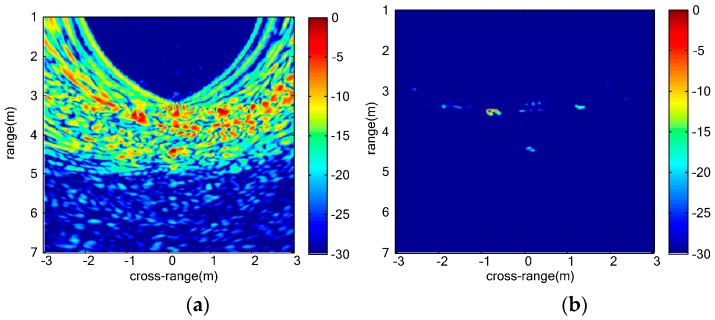
Images of multiple stationary humans: (**a**) UWB MIMO image at certain slow time; and (**b**) vital-sign-enhanced image.

**Figure 12:**
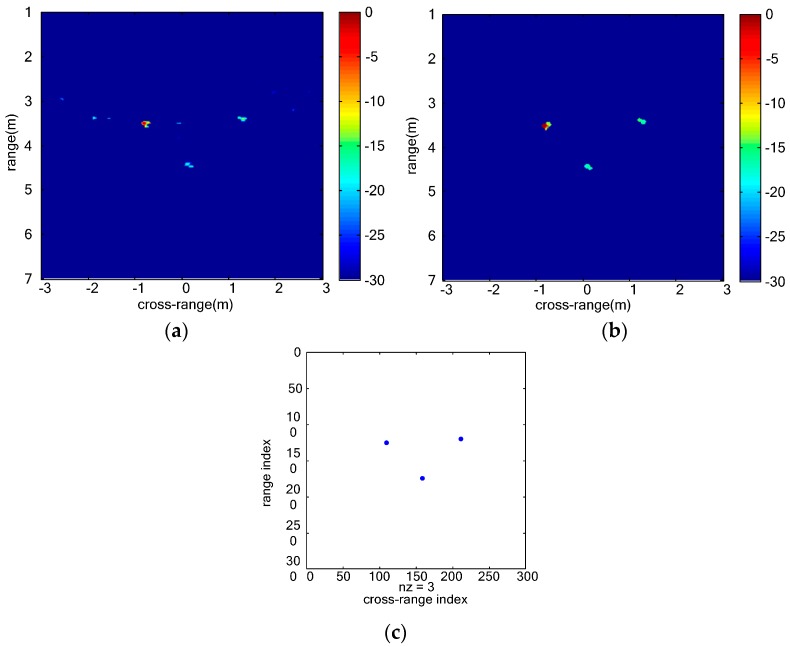
Detection results of multiple stationary humans: (**a**) CFAR image; (**b**) morphological filtering result; and (**c**) clustering result.

The measurement scenario of three stationary humans is shown as Figure 10. Figure 10a shows the locations of three vital signs. UWB MIMO image at a certain slow time is shown as Figure 11a. The images of three vital signs are severely blurred due to mutual interference of strong sidelobes. The echo of the far-end human target is weak compared with the other two nearby humans due to the shadow effect. Sidelobes of the two nearby humans integrate with each other to form strong clutters. The clutters caused by sidelobes are effectively suppressed and the SCR of vital signs improves remarkably by the proposed vital-sign-enhanced imaging algorithm. The SCRs of human target 1 in MIMO image and vital-sign-enhanced image are 17.5732 dB and 56.7852 dB, respectively. The detection results of three stationary humans are given in Figure 12. We can easily discriminate three human targets. Similarly, the measured target locations accord with the real target positions. 

## 6. Conclusions

SFCW signaling combined with MIMO processing enables the radar to have high range resolution as well as high cross-range resolution. In this paper, a vital signs model of UWB MIMO radar is employed, and then a novel automatic detection and localization method is presented. This method fully utilizes the two-dimensional resolution of UWB MIMO radar to improve the space separation between the humans and clutters and among multiple humans. Micro motion of the human body is utilized in the enhanced imaging to suppress the sidelobes and clutters, thus the mutual interferences among multiple humans can be suppressed. A procedure including CFAR, morphological filtering and clustering is proposed to detect and localize multiple humans automatically in low SCR conditions. The simulation and experimental results verify the effectiveness of the proposed method. Especially, in through-the-wall experiments of multiple humans detection, three adjacent humans can be discriminated and detected. However, additional efforts are required to study the estimation of wall parameters in through-the-wall image formation. More experiments in realistic ruins conditions should also be considered to test the performance of the UWB MIMO system and the proposed processing method in complex scenarios.

## Figures and Tables

**Table 1:** Parameters of the UWB MIMO radar.

Parameters	Value
Bandwidth	40–4400 MHz
Frequency step	5 MHz
Pulse repeated frequency (PRF)	113 Hz
Unambiguous range	≥30 m
Transmitting power	≥10 dBm
Sensitivity of receiver	−90 dBm
Dynamic range of receiver	≥90 dB
